# Machine learning insights into scapular stabilization for alleviating shoulder pain in college students

**DOI:** 10.1038/s41598-024-79191-8

**Published:** 2024-11-18

**Authors:** Omar M. Mabrouk, Doaa A. Abdel Hady, Tarek Abd El-Hafeez

**Affiliations:** 1 Basic Science for Physical Therapy, Deraya University, EL-Minia, Egypt; 2 Department of Physical Therapy for Women’s Health, Faculty of Physiotherapy, Deraya University, EL-Minia, Egypt; 3 Department of Computer Science, Faculty of Science, Minia University, EL-Minia, Egypt; 4 Computer Science Unit, Deraya University, EL-Minia, Egypt

**Keywords:** Machine learning, Scapular stabilization exercises, Non-specific Shoulder Pain, College Students, Health care, Health occupations, Medical research, Risk factors, Signs and symptoms, Computer science, Information technology, Scientific data

## Abstract

Non-specific shoulder pain is a common musculoskeletal condition, especially among college students, and it can have a negative impact on the patient’s life. Therapists have used scapular stabilization exercises (SSE) to enhance scapular control and mobility. This study investigates the prediction of the impact of scapular stability exercises in treating non-specific shoulder pain, leveraging advanced machine learning techniques for comprehensive evaluation and analysis. Using a diverse range of regression models, including Gamma Regressor, Tweedie Regressor, Poisson Regressor, and others, the study examines the relationship between the effectiveness of various exercises and their impact on shoulder pain management. Furthermore, the study employs optimization techniques, such as Hyperopt, scikit-optimize, optunity, GPyOpt, and Optuna, to fine-tune the exercise protocols for optimal outcomes. The results reveal that scapular stabilization exercises, when optimized using machine learning algorithms, significantly contribute to reducing shoulder pain in college students. Among the optimization techniques, scikit-optimize demonstrated the best performance, resulting in a mean squared error of 0.0085, a mean absolute error of 0.0712, and an impressive R2 score of 0.8501. This indicates that the scikit-optimize approach yielded the most accurate predictions and effectively captured the relationship between the exercises and shoulder pain management. The findings highlight the critical role of scapular stabilization exercise interventions in ameliorating non-specific shoulder pain and underscore the potential of machine learning techniques in optimizing therapeutic strategies for musculoskeletal health management. The utilization of scikit-optimize, in particular, showcases its effectiveness in fine-tuning the exercise protocols for optimal outcomes. The study’s results serve as a crucial stepping stone in developing personalized rehabilitation programs for non-specific shoulder pain, emphasizing the importance of integrating machine learning methodologies in the assessment and treatment of musculoskeletal disorders among college students.

## Introduction

Shoulder pain is a prevalent condition in therapeutic practice, with an incidence rate of approximately 10 per 1000 patients in medical care^1,2^ and a prevalence of 12% in physiotherapy settings^3^. It is a common musculoskeletal issue that can significantly impact daily activities and quality of life^4^. Non-specific shoulder pain, characterized by persistent discomfort without a clear underlying pathology, presents a complex challenge for clinicians. This condition often arises from a combination of factors such as poor posture, repetitive movements, muscle imbalances, and inadequate scapular stabilization, all of which contribute to functional limitations^5,6^.

Scapular stabilization exercises (SSE) have been widely recognized as an effective intervention for managing non-specific shoulder pain. These exercises aim to enhance shoulder function by improving the balance of the scapular muscles, correcting scapular dyskinesis, and restoring proper force coupling^7,8^. Scapular stabilization can result in significant improvements in pain reduction, muscle strength, and overall shoulder function^9^. However, despite their demonstrated efficacy, there remains a critical need to optimize the implementation of these exercises. Variations in patient responses and the challenges in individualizing exercise programs highlight the necessity for further investigation into the most effective strategies for different populations^10,11^.

While scapular stabilization exercises are generally effective, their impact can vary significantly across individuals and populations. This variability highlights a critical need for more precise, data-driven approaches that can objectively evaluate which patients are most likely to benefit from these interventions. In certain cases, these exercises may be less effective or even counterproductive, underscoring the importance of using machine learning to identify patient characteristics that predict positive outcomes. By tailoring treatment in this way, clinical practice can move toward a more personalized and efficient approach to rehabilitation^12,13^.

In this context, the integration of machine learning techniques offers a promising avenue for advancing the evaluation and optimization of scapular stabilization exercises. Machine learning has already demonstrated its potential in analyzing complex data sets and providing insights that can improve clinical decision-making. By applying these techniques to musculoskeletal disorders, researchers can uncover biomechanical patterns and relationships that may otherwise remain obscured, ultimately leading to more effective and personalized treatment strategies^14,15^.

Given the increasing incidence of musculoskeletal issues among college students—attributed to sedentary lifestyles and academic demands^16^—it is particularly important to investigate the role of scapular stabilization exercises in this population. The current study aims to explore the use of machine learning to enhance the implementation of these exercises for non-specific shoulder pain in college students. By integrating computational analytics with traditional rehabilitation approaches, the study seeks to improve patient outcomes and contribute to the broader understanding of musculoskeletal health management.

### Problem statement

Non-specific shoulder pain is a common and debilitating condition, particularly prevalent among college students due to factors such as poor posture and prolonged sedentary behavior. While scapular stabilization exercises (SSE) are widely recognized as an effective intervention for improving scapular control and reducing shoulder pain, there is a significant challenge in optimizing their implementation to account for individual patient differences. Traditional approaches to prescribing these exercises often rely on subjective measures and general protocols, which may not yield consistent results across diverse populations. Furthermore, there is a lack of a systematic, data-driven approach to personalize SSE interventions to maximize their therapeutic outcomes. This research aims to address these gaps by leveraging advanced machine learning techniques to predict and optimize the effectiveness of scapular stabilization exercises in treating non-specific shoulder pain among college students.

### Research question

How can machine learning techniques be applied to optimize scapular stabilization exercises for the treatment of non-specific shoulder pain, and what machine learning models and optimization techniques are most effective in predicting treatment outcomes among college students?

### Research gap

While the effectiveness of scapular stabilization exercises in managing shoulder pain has been well-documented, the current literature lacks comprehensive studies that apply machine learning to enhance and individualize these interventions. Previous research has primarily focused on standardized exercise regimens, which may not account for the variability in patient responses. Furthermore, there is limited exploration of how different machine learning techniques, such as regression models and optimization algorithms, can be used to fine-tune exercise protocols for optimal outcomes. This study addresses this gap by integrating machine learning into the evaluation and optimization of scapular stabilization exercises, offering a more personalized and data-driven approach to shoulder pain management.

### Contributions

The main contributions of this research can be summarized as follows:

- Application of Machine Learning for SSE Optimization: This study utilizes a range of regression models, including Gamma Regressor, Tweedie Regressor, and Poisson Regressor, to predict the outcomes of scapular stabilization exercises based on patient-specific data. These models provide a more objective and precise way of evaluating the effectiveness of SSE interventions.
- Optimization of Exercise Protocols: Through the use of hyperparameter optimization algorithms such as Hyperopt, Scikit-Optimize, and Optuna, this research identifies the optimal exercise parameters for individual patients, significantly improving the personalization of treatment plans.
- Evaluation of Machine Learning Models: The study compares the performance of different machine learning and optimization techniques for predicting patient outcomes. Among these, Scikit-Optimize demonstrated the best performance, achieving a high R2 score of 0.8501, indicating its effectiveness in capturing the relationship between SSE interventions and pain reduction.
- Advancing Personalized Rehabilitation: By integrating machine learning into the rehabilitation process, this study pioneers a personalized approach to treating non-specific shoulder pain in college students, offering a data-driven methodology that could enhance patient outcomes in clinical practice.
- Contribution to Musculoskeletal Health Management: This research underscores the potential of machine learning in transforming the assessment and treatment of musculoskeletal disorders, providing a framework that could be extended to other therapeutic interventions beyond scapular stabilization.

## Materials and methods

### Trial design

This trial was designed as an observational and cross-section study. The study was approved by the Ethical Committee at Deraya University, (No: 19/2023). According to the ethical standards of the Declaration of Helsinki^17^, This study complies with the principles for human research. Each patient signed a written consent form after being given a thorough description of the trial. The study was conducted at Deraya outpatient clinic from April 20, 2023, till July 25, 2023.

### The sample size

To minimize the risk of Type II errors (failing to detect a statistically significant difference when one exists), a priori sample size calculation was conducted utilizing G*Power software, specifically tailored for the Wilcoxon-Mann-Whitney test^18,19^). This computation was informed by the following statistical parameters:

- Effect Size (d): 0.5, indicating a moderate expected difference between groups.
- Effect Size for Wilcoxon-Mann-Whitney (dz): 0.5, consistent with the chosen effect size.
- Power (1-β): 0.95, aiming for high confidence in detecting significant differences.
- Significance Level (α): 0.05 (two-tailed), maintaining a balanced approach to error control.

The calculation revealed that a minimum sample size of 40 participants was necessary to ensure that the confidence intervals for the agreement between any two measures would be sufficiently precise, approximating half a standard deviation of their discrepancies.

Notably, the actual dataset for this study comprises 85 patients. This substantial excess in sample size provides several assurances:

1. Enhanced Statistical Power: The study is well-equipped to detect statistically significant differences between measures with a high degree of confidence.
2. Reliability and Generalizability of Findings: The larger sample size contributes to more reliable and genuine results, enhancing the study’s external validity and the potential for generalizing the conclusions to similar populations.
3. Robustness Against Type II Errors: The ample sample size effectively mitigates the risk of failing to identify significant effects, should they exist, thereby bolstering the study’s overallMethodological rigor.

### Participants

Eighty-five college students who complained of non-specific unilateral shoulder pain lasting > 6 weeks were included in this study based on the following criteria: their ages ranged from 18 to 25 years, after consulting orthopedic surgeon diagnosis confirmation as if the patients exhibited at least 2 of the following (1) painful arc during flexion or abduction from 60 to 120 degree(2) a positive Neer test(3) Painful resisted external rotation, abduction. (4) Type 1 and 2 scapular dyskinesia.

### Exclusion criteria

MRI or ultrasound confirmation of torn rotator cuff tendons (partial or full-thickness tear), inability to lift the arm to 90 of abduction, a cervical range of motion that reproduced shoulder pain, pain below the elbow indicative of cervical or nerve pathologies, past shoulder surgery, and glenohumeral joint arthritis, as indicated in the report, Acute trauma (fractures, traumatic dislocations), inflammatory arthritis.

### Outcome measures

#### Assessment of pain

The visual analog scale (VAS) is a one-dimensional measure of pain intensity that is used to compare the pain severity of patients pre and post-scapular stabilization treatment. The VAS has excellent test-retest reliability (*r* = .94) and correlates highly with other pain measurement tools^20^.

#### Assessment Acromion-humeral distance (AHD)

Two ultrasound images were collected with the arm at rest and 90° active abduction pre and post-exercise sessions with the subject in a sitting position with the elbow flexed 90° supported on his thigh. The outlet of the sub-acromion space was measured with 2-D ultrasound images via the AHD. The AHD is defined as the shortest distance between the acromion and the humerus. An ultrasound unit (mindray DP 10) with a 10 MHz linear ultrasound transducer was utilized. The placement of the ultrasound transducer was standardized, with its location on the posterior to the middle portion of the acromion in the coronal plane, with the transducer placed parallel to the flat superior aspect of the acromion so that both the acromion and humerus were visualized^21^. Ultrasound as an imaging modality is less costly and more practical than MRI and has established concurrent validity with radiographic AHD measures (*r* = .77–0.85)^22,23^.

### Interventions

#### Scapular stabilization exercise intervention

From the sitting position shoulder shrug at 0° abduction and 30° abduction, scapular retraction W exercises against handheld theraband, external rotation with elbow 90° flexion, and external rotation with forward flexion against handheld theraband. From the Prone laying position external rotation at 90° abduction and elbow at 90° using a dumbbell, All the exercises were performed10 repetitions/set, 3 sets/day, and 3 days/week for 4 weeks^8^.

### Related work

In this section, we embark on a comprehensive journey through the landscape of related work. We explore a collection of seminal studies that have diligently examined the impact of scapular exercise interventions on shoulder conditions, shedding light on the effectiveness of these exercises in managing ailments such as subacromial impingement syndrome (SIS) and non-specific shoulder pain. These studies have collectively enriched our understanding of the pivotal role that scapular stabilization exercises play in shoulder dysfunction rehabilitation and the alleviation of related discomfort. Scapular dyskinesis and weakness have been increasingly recognized as contributing factors to shoulder impingement syndrome (SIS). Several studies have investigated the effects of scapular-focused exercises in the treatment of SIS. Table 1 summarizes the key findings from several relevant articles on this topic.

**Table 1:** Summary of the key findings from several relevant articles SIS.

Study	Summary
Ravichandran, H., et al.,^24^	Articles included in this review demonstrated effects supporting the use of scapular exercise as an intervention for impingement syndrome of the shoulder joint. The conclusion of this review suggests that the best clinical outcome for improving subacromial dysfunction would occur with the implementation of scapular stabilization exercises either solely or as a part of shoulder pain rehabilitation.
Mohamed, H. et al.,^25^	Results showed that there is an excellent sensitivity and positive predictor of the use of scapular training in patients with subacromial impingement syndrome associated with shoulder pain.
Lee, J., et al.,^26^	The study examined the relationship between forward scapular posture and related predictor variables. The findings demonstrated moderate to high correlations between forward scapular posture and non-specific shoulder pain by 78% of the forward scapular posture in a simple regression.
Turgut, E.,et al.,^27^	Progressive scapular stabilization exercises provide decreased disability and pain severity in patients with shoulder pain.
Moezy A. et al.,^28^	Exercise prescription to enhance scapular stabilization during the SIS rehabilitation. The scapular stabilization-based exercise intervention was successful at increasing the shoulder range of abduction and external rotation, decreasing forward head and shoulder postures, and Pectoralis minor flexibility. This study supports the basis for scapular stabilization-based exercise therapy in the rehabilitation of SIS. Also, be noted that exercise therapy is effective as a treatment for the reduction of pain in these patients.

## Methodology

### Regression techniques

Table 2 presents a comprehensive overview of diverse regression models and techniques, along with their associated regularizations and cross-validation strategies, commonly employed in the context of machine learning and statistical analysis. These models serve as fundamental building blocks in the evaluation and prediction of complex relationships within datasets, enabling researchers and practitioners to address a wide array of analytical challenges and optimize predictive performance. By offering a detailed description of each model’s characteristics, suitable data types, advantages, and potential limitations, this table serves as a valuable guide for selecting appropriate regression methodologies tailored to specific research objectives and dataset requirements. From traditional linear regression techniques to advanced ensemble methods and robust regression algorithms, the table outlines the key features and applications of each model, facilitating a comprehensive understanding of the diverse tools available for data analysis and predictive modeling.

**Table 2:** Summary of the regression techniques used in the study.

Model	Description	Regularization	Cross-Validation	Suitable for	Advantages	Disadvantages
GammaRegressor ^29^	Gamma distribution-based regression model	None	No	Non-negative, right-skewed data	Appropriate for specific data types	Limited applicability to some data
TweedieRegressor ^30^	Tweedie distribution-based regression model	None	No	Normal and non-normal data	Handles both normal and non-normal data	Complex model selection due to power
PoissonRegressor ^31^	Poisson distribution-based regression model	None	No	Count data with non-negative values	Suitable for counting data	Assumes equidispersion (variance = mean)
LassoLarsCV ^32^	Lasso regression with cross-validated alpha selection	L1 (Lasso)	Yes	Feature selection, sparsity	Automatic alpha selection for L1 reg.	Less interpretable compared to linear
LarsCV ^33^	Least Angle Regression (LARS) with cross-validation	None	Yes	High-dimensional data	Efficient with high-dimensional data	May select too many features for small
ElasticNetCV ^34^	Elastic Net regression with cross-validated alpha	L1 and L2 (Elastic Net)	Yes	L1 and L2 regularization	Combines L1 and L2 reg. for flexibility	Complex tuning with two hyperparameters
LassoCV ^35^	Lasso regression with cross-validated alpha selection	L1 (Lasso)	Yes	Feature selection, sparsity	L1 regularization for feature selection	This may lead to feature selection bias
LassoLarsIC ^36^	Lasso LARS (Least Angle Regression) with AIC/BIC	L1 (Lasso)	No	Automatic alpha selection	Automatic alpha selection with criteria	May overfit with large feature sets
RidgeCV ^37^	Ridge regression with cross-validated alpha	L2 (Ridge)	Yes	Reducing multicollinearity	L2 regularization for multicollinearity	Does not perform feature selection
BayesianRidge ^38^	Bayesian Ridge regression using Bayesian methods	L2 (Ridge)	No	Multicollinearity, model uncertainty	Handles multicollinearity, uncertainty	Requires prior knowledge of hyperparameters
Orthogonal MatchingPursuitCV ^39^	Orthogonal Matching Pursuit with cross-validated regularization	None	Yes	Sparse models	Efficient for sparse models	May not perform well with correlated
BaggingRegressor ^40^	Ensemble method using multiple base models	None	No	Reducing overfitting, model stability	Reduces overfitting and enhances stability	May not improve performance with strong
Ridge ^41^	Ridge regression with a fixed alpha parameter	L2 (Ridge)	No	Reducing multicollinearity	Requires manual tuning of alpha	
LinearRegression ^42^	Simple linear regression with no regularization	None	No	Simple cases	Easy to interpret and suitable for simple	Sensitive to outliers and multicollinear
Lars ^43^	Least Angle Regression (LARS) without regularization	None	No	High-dimensional data	Efficient for high-dimensional data	May select too many features for small
TransformedTargetRegressor ^44^	Applies a power or log transformation to the target	None	No	Handling heteroscedasticity	Handles heteroscedasticity and non-linear	Requires knowledge of transformation
SGDRegressor ^45^	Stochastic Gradient Descent for regression	L1 or L2 (Optional)	No	Large datasets, online learning	Suitable for large datasets and online	Sensitive to hyperparameters and noisy
RandomForest Regressor ^46^	Ensemble method using random forests	None	No	Complex relationships, interactions	Handles complex relationships and inter	May overfit with deep trees and small
ExtraTrees Regressor ^47^	Ensemble method using extremely randomized trees	None	No	Reducing overfitting	Reduces overfitting with random feats.	This may lead to a loss of interpretability
HuberRegressor ^48^	Robust regression with combined L1 and L2 loss	L1 (Huber)	No	Resistant to outliers	Resistant to outliers while efficient	Requires tuning of the ‘epsilon’ parameter

### Dataset characteristics

The provided data represents a table with various columns containing information about individuals, including their sex, age, weight, BMI (Body Mass Index), Pretreatment SAS (Self-Appraisal Scale) zero, Pretreatment SAS 90, and Posttreatment SAS 90 scores. The dataset contains the following columns:

- **Sex**: This variable indicates the gender or biological sex of the patient and can be categorized as male or female. In some cases, it might also include options for other gender identities.
- **Age**: Age represents the patient’s chronological age, typically measured in years. It is a fundamental demographic variable used to understand how age might impact various health-related factors.
- **Weight**: This variable denotes the patient’s body weight, often measured in kilograms (or pounds in some regions). Weight is crucial for assessing overall health and monitoring changes during treatment.
- **BMI (Body Mass Index)**: BMI is a calculated value that relates to a person’s weight and height. It is used to assess whether a person is underweight, normal weight, overweight, or obese. The formula for calculating BMI is (weight in kilograms) / (height in meters squared) or (weight in pounds) / (height in inches squared) multiplied by 703.
- **Pretreatment SAS Zero**: The SAS (Self-Rating Anxiety Scale) is a psychological assessment tool used to measure a person’s level of anxiety. “Pretreatment SAS Zero” might refer to the patient’s anxiety score before any treatment or intervention, with “zero” indicating the baseline measurement.
- **Pretreatment SAS 90**: Similar to “Pretreatment SAS Zero,” this variable represents the patient’s anxiety score before any treatment or intervention, but the “90” suggests it could be a specific time point or measurement related to the treatment process. The SAS scale is typically scored out of 100.
- **VAS pre**: VAS (Visual Analog Scale) is a tool used to measure subjective experiences, such as pain or discomfort. “VAS pre” likely refers to the patient’s self-reported assessment on this scale before any treatment.
- **VAS post**: Similar to “VAS pre,” this variable represents the patient’s self-reported assessment on the Visual Analog Scale after the treatment or intervention has been administered.
- **Posttreatment SAS 90**: This variable denotes the patient’s anxiety score after receiving treatment or intervention, with “90” potentially indicating a specific time point or measurement related to the post-treatment phase. It assesses the level of anxiety following the treatment.

This dataset contains information related to (85) individuals’ characteristics, including their demographics (sex and age), physical attributes (weight and BMI), and self-appraisal scores before and after some form of treatment or intervention. The specific nature of the treatment or intervention and the meaning of the self-appraisal scores (SAS) would require additional context to fully understand the dataset’s purpose and analysis.

**Fig. 1:**
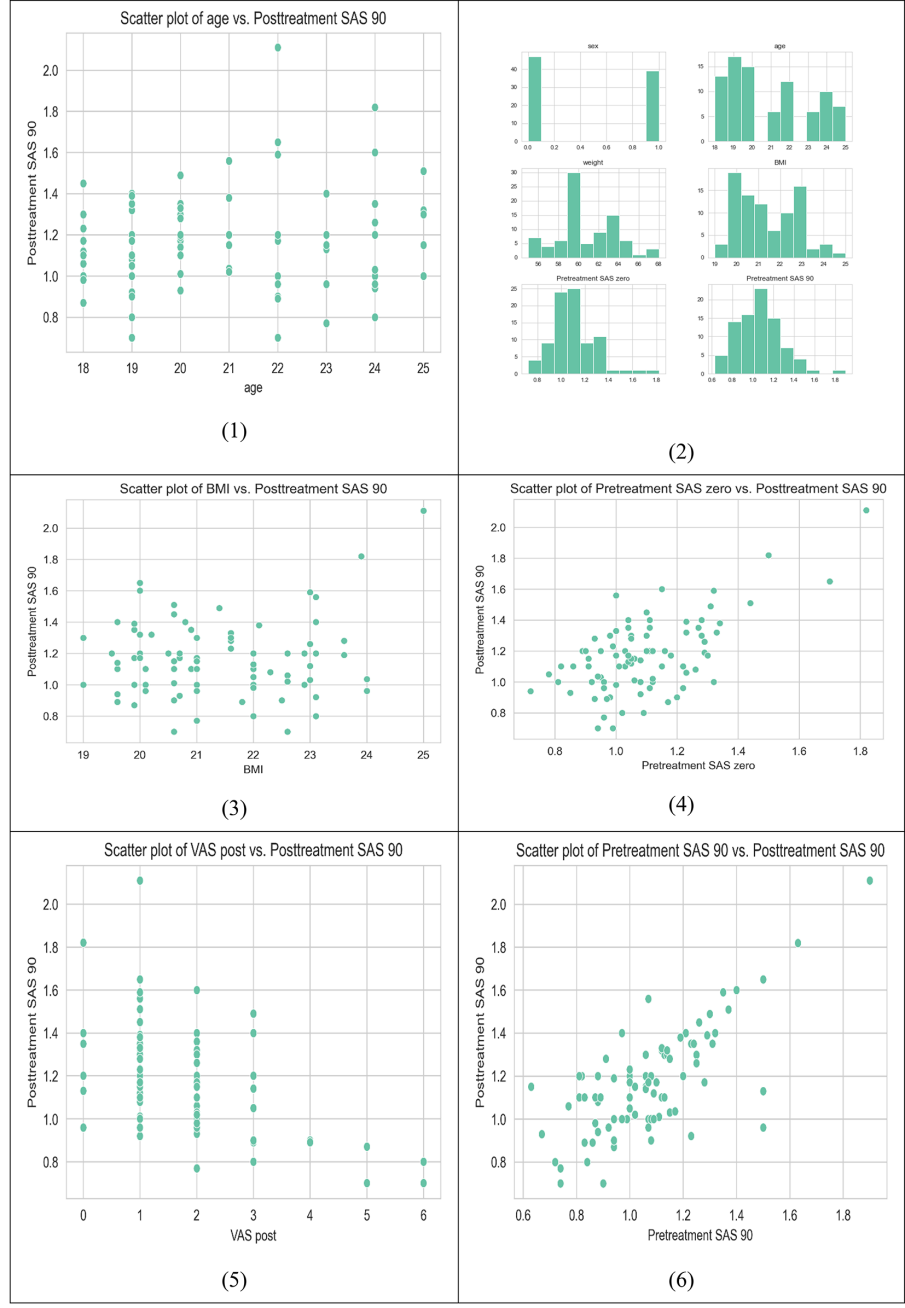
Correlation between dataset features.

Table 3; Fig. 1 provide a comprehensive summary of various features, including descriptive statistics that offer insights into the central tendencies, variability, and distribution of each feature within the dataset. These features encompass a range of information, such as demographic attributes (sex, age), physical characteristics (weight (Kg), BMI), and self-appraisal scores both before and after a treatment or intervention (Pretreatment SAS zero, Pretreatment SAS 90). By examining the mean, median, standard deviation, and percentiles (25th, 50th, and 75th) for each feature, we can gain a better understanding of the dataset’s characteristics and the distribution of the variables. This table serves as a valuable reference point for analyzing and interpreting the dataset, shedding light on the central tendencies and variability within the dataset’s key attributes.

**Table 3:** Descriptive statistics of the dataset features.

Feature	mean	median	std_dev	min	25%	50%	75%	max
sex	0.45	0.00	0.50	0.00	0.00	0.00	1.00	1.00
age	20.96	20.00	2.26	18.00	19.00	20.00	23.00	25.00
Weight (Kg)	60.69	60.00	2.96	55.00	59.00	60.00	63.00	68.00
BMI	21.36	21.00	1.36	19.00	20.10	21.00	22.60	25.00
Pretreatment SAS zero	1.10	1.06	0.18	0.72	0.98	1.06	1.20	1.82
Pretreatment SAS 90	1.07	1.07	0.22	0.63	0.91	1.07	1.19	1.90
VAS pre	5.45	5.00	1.16	4.00	5.00	5.00	6.00	8.00
VAS post	1.76	2.00	1.20	0.00	1.00	2.00	2.00	6.00

Table 4 shows the relationship between the numerical variables in the dataset. Each row and column in the matrix represents a continuous variable, and Pearson’s R-value corresponding to that row and column reflects the strength and direction of the correlation between the variables. Most qualities are significantly connected, according to our observations. This matrix provides an in-depth look at the correlations between various attributes, with each attribute listed on both the rows and columns. The numbers in the rows and columns show the correlation coefficient between the two traits, with a coefficient close to 1 representing a high positive correlation, a coefficient close to -1 representing a strong negative correlation, and a coefficient close to 0 representing no association.

**Table 4:** The correlation heat map of the proposed framework.

	sex	weight	BMI	Pretreatment SAS zero	Pretreatment SAS 90	VAS pre	VAS post	Posttreatment SAS 90
sex	1.000	0.007	-0.071	0.081	0.042	-0.102	0.038	0.041
weight	0.007	1.000	0.540	-0.089	-0.049	0.119	-0.044	0.017
BMI	-0.071	0.540	1.000	0.082	0.090	0.040	-0.031	0.144
Pretreatment SAS zero	0.081	-0.089	0.082	1.000	0.616	-0.423	-0.278	0.598
Pretreatment SAS 90	0.042	-0.049	0.090	0.616	1.000	-0.770	-0.445	0.709
VAS pre	-0.102	0.119	0.040	-0.423	-0.770	1.000	0.478	-0.410
VAS post	0.038	-0.044	-0.031	-0.278	-0.445	0.478	1.000	-0.504
Posttreatment SAS 90	0.041	0.017	0.144	0.598	0.709	-0.410	-0.504	1.000

### Preliminaries

**Fig. 2:**
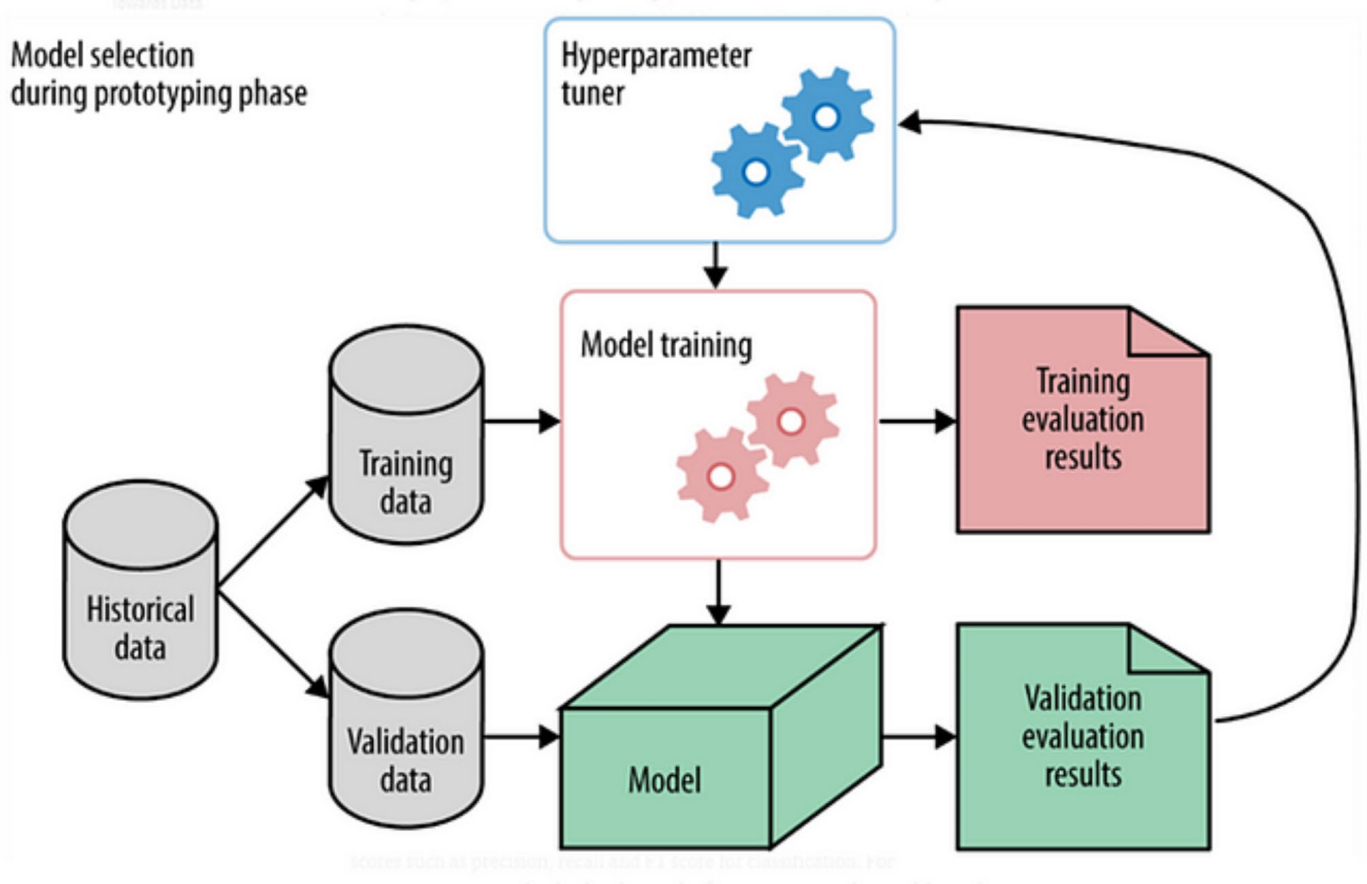
Hyperparameter Tuner^52^.

Hyperparameter tuning involves the search for an optimal set of parameters that can significantly enhance the precision and accuracy of a model^49–51^. This process is known to be one of the most challenging aspects of developing machine learning models. Predicting the ideal hyperparameters during the initial model construction is exceedingly difficult. The fundamental objective of hyperparameter tuning is to identify the optimal configuration for a model’s parameters, thereby achieving a superior performance level, as illustrated in Fig. 2.

The depicted figure illustrates the separation of the “hyperparameter tuner” from the model, highlighting that the tuning occurs before the model training phase. The output of the tuning process is the identified optimal values of hyperparameters, which are subsequently utilized during model training.

Hyperparameter optimization constitutes a vital phase in the training of Machine Learning models. Given the numerous parameters that require optimization, coupled with extended training durations and the necessity of implementing multiple folds to prevent information leakage, the process can become a laborious undertaking.

### Hyperopt

**Fig. 3:**
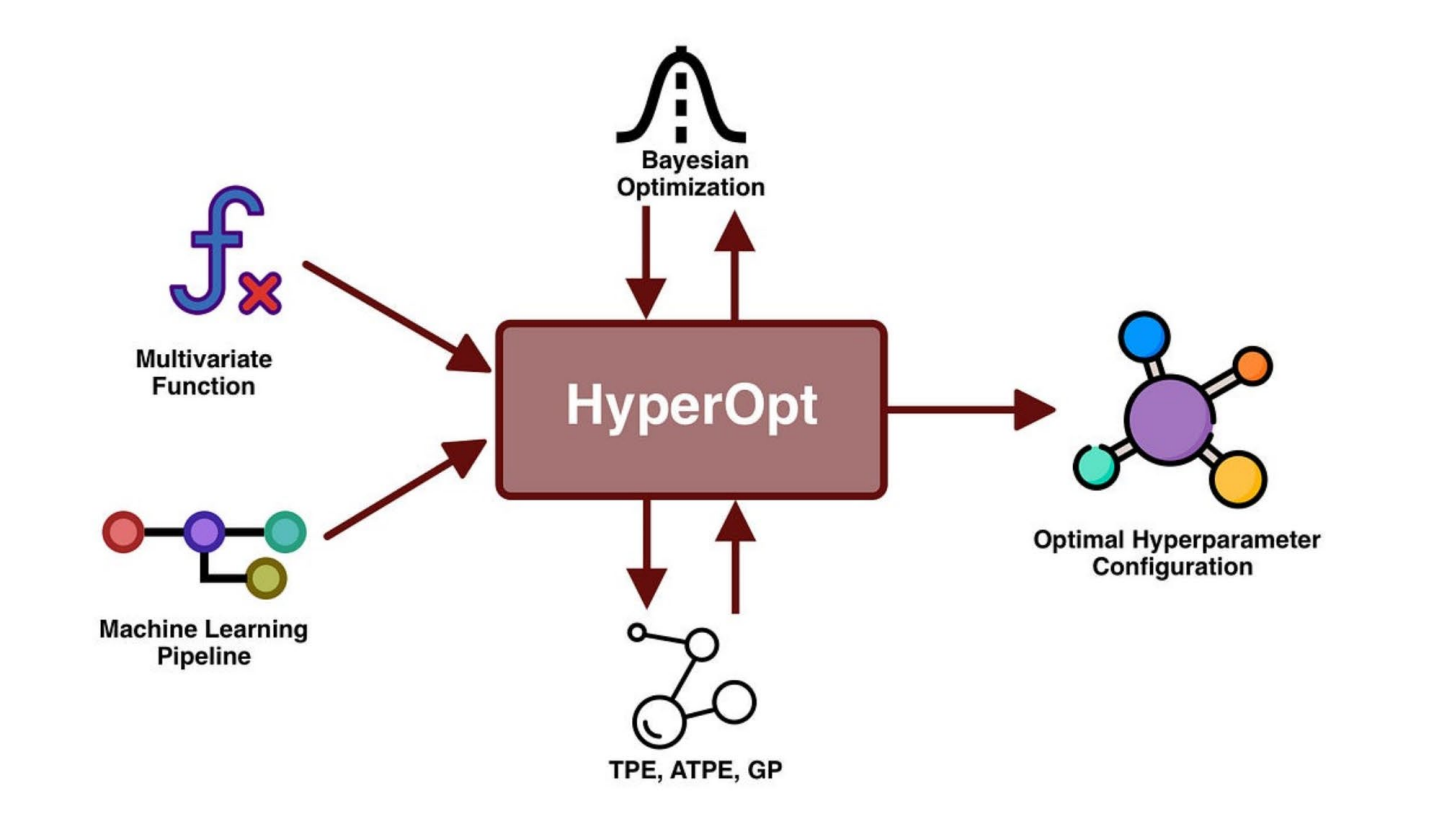
A representative architecture of HyperOpt^53^.

Hyperopt stands as a popular open-source Python library designed for the optimization of hyperparameters in machine learning models. Created by James Bergstra, Brent Komer, and a team of contributors, Hyperopt is specifically tailored for hyperparameter optimization, employing a Bayesian optimization technique. Its primary objective lies in the identification of the most optimal set of hyperparameters, thereby maximizing the overall performance of machine learning models, as depicted in Fig. 3.

### Key features and concepts

- **Bayesian Optimization:** Hyperopt employs Bayesian optimization, a probabilistic model-based optimization technique that efficiently explores and exploits the hyperparameter space. This approach uses a surrogate model to estimate the objective function and its uncertainty, enabling the optimizer to make informed decisions about which hyperparameters to explore next.
- **Tree-structured Parzen Estimator (TPE):** Hyperopt utilizes the TPE algorithm to guide the search for optimal hyperparameters. TPE is known for its efficiency in finding the best configurations by effectively balancing exploration and exploitation.
- **Parallel and Distributed Optimization:** Hyperopt supports parallel and distributed optimization, allowing users to harness the power of multi-core processors and distributed computing clusters. This can significantly speed up the hyperparameter search process.
- **Extensive Search Space:** Hyperopt can handle a wide range of hyperparameters, making it suitable for optimizing a variety of machine learning models, including deep learning neural networks, support vector machines, and gradient boosting machines.

#### Use cases

Hyperopt is commonly applied to fine-tune hyperparameters in machine learning models, improving their performance and generalization. It is widely used in the fields of data science, deep learning, and machine learning research to automate and optimize the model selection process.

### Scikit-optimize - forest_minimize

#### Description

Scikit-Optimize, often referred to as **skopt**, is a Python library designed for hyperparameter optimization. The **forest_minimize** function in Scikit-Optimize^54^ is a specific optimizer that utilizes Bayesian optimization techniques.

### Key features and concepts

- **Surrogate Models:** Scikit-Optimize, including **forest_minimize**, employs surrogate models such as Gaussian processes to approximate the objective function. These surrogate models are computationally efficient and enable the optimizer to make informed decisions about where to sample next.
- **Acquisition Functions:** The optimizer relies on acquisition functions (e.g., Probability of Improvement or Expected Improvement) to guide the search. These functions help strike a balance between exploring uncharted regions of the hyperparameter space and exploiting regions that appear promising.
- **Customizable Search Space:** Scikit-Optimize offers flexibility in defining the search space for hyperparameters. Users can specify both continuous and categorical hyperparameters and set their bounds.

#### Use cases

Scikit-Optimize, including **forest_minimize**, is a valuable tool for optimizing machine learning models, as well as other applications involving hyperparameter tuning. It applies to various domains, including natural language processing, computer vision, and scientific research.

### Optunity

#### Description

Optunity^55^ is a Python library for hyperparameter optimization and model selection. It combines various optimization techniques, such as grid search, random search, and Bayesian optimization, to find the best hyperparameters for a given problem.

### Key features and concepts

- **Fitness Function:** Optunity is highly customizable and works with user-defined fitness functions. These fitness functions encapsulate the specific problem that needs to be optimized. Optunity aims to either maximize or minimize these functions.
- **Versatility:** Optunity is not limited to machine learning model hyperparameter tuning. It can be applied to a wide range of optimization problems, making it a versatile tool for researchers and practitioners.
- **Hybrid optimization:** Optunity can employ different optimization algorithms, including grid search, random search, and Bayesian optimization, to explore the hyperparameter space. This adaptability allows it to strike a balance between computational efficiency and accuracy.

#### Use cases

Optunity is used in applications that require optimization across various domains, including machine learning, scientific research, and engineering. It can be applied to optimize model hyperparameters, experimental setups, and more.

### GPyOpt

#### Description

GPyOpt is a Python library designed for Bayesian optimization. It leverages Gaussian processes as surrogate models to approximate the objective function. It is particularly useful for optimizing complex and expensive-to-evaluate functions.

### Key features and concepts

- **Gaussian processes:** GPyOpt relies on Gaussian processes to model the objective function^56^. These probabilistic models provide not only point estimates but also uncertainty estimates, making them well-suited for black-box optimization problems.
- **Acquisition functions:** Similar to other Bayesian optimization libraries, GPyOpt employs acquisition functions (e.g., Probability of Improvement) to determine which points to sample next. These functions consider the trade-off between exploration and exploitation.
- **Probabilistic optimization:** GPyOpt excels in handling noisy and uncertain objective functions. It estimates both the mean and variance of the objective function, allowing for more informed decisions during optimization.

#### Use cases

GPyOpt is commonly used in scenarios where the objective function is expensive to evaluate, such as optimizing hyperparameters for deep learning models, engineering design, and scientific experiments.

### Optuna

#### Description

Optuna^57^ is a Python library known for its simplicity, flexibility, and efficiency in automated hyperparameter optimization. It was developed by the Preferred Networks team to facilitate the optimization of machine learning and deep learning models.

### Key features and concepts

- **Tree-structured Parzen Estimator (TPE):** Optuna uses TPE, a Bayesian optimization algorithm, to guide the hyperparameter search. TPE efficiently balances exploration and exploitation in the search process.
- **Study and Trial:** In Optuna, optimization problems are organized into studies, each of which contains multiple trials. Each trial represents an attempt to optimize the objective function by sampling different hyperparameter configurations.
- **Pruning:** Optuna employs pruning techniques to discard unpromising trials, saving computational resources. This is especially useful when optimizing over a large search space.
- **Parallel and Distributed Optimization:** Optuna supports parallel and distributed optimization, enabling users to harness multiple processors or even entire computing clusters to accelerate the optimization process.

#### Use cases

Optuna is extensively used for automating the hyperparameter tuning process in machine learning and deep learning applications. Its simplicity and efficiency make it accessible to both beginners and experts in the field of artificial intelligence and data science.

### The proposed framework

**Fig. 4:**
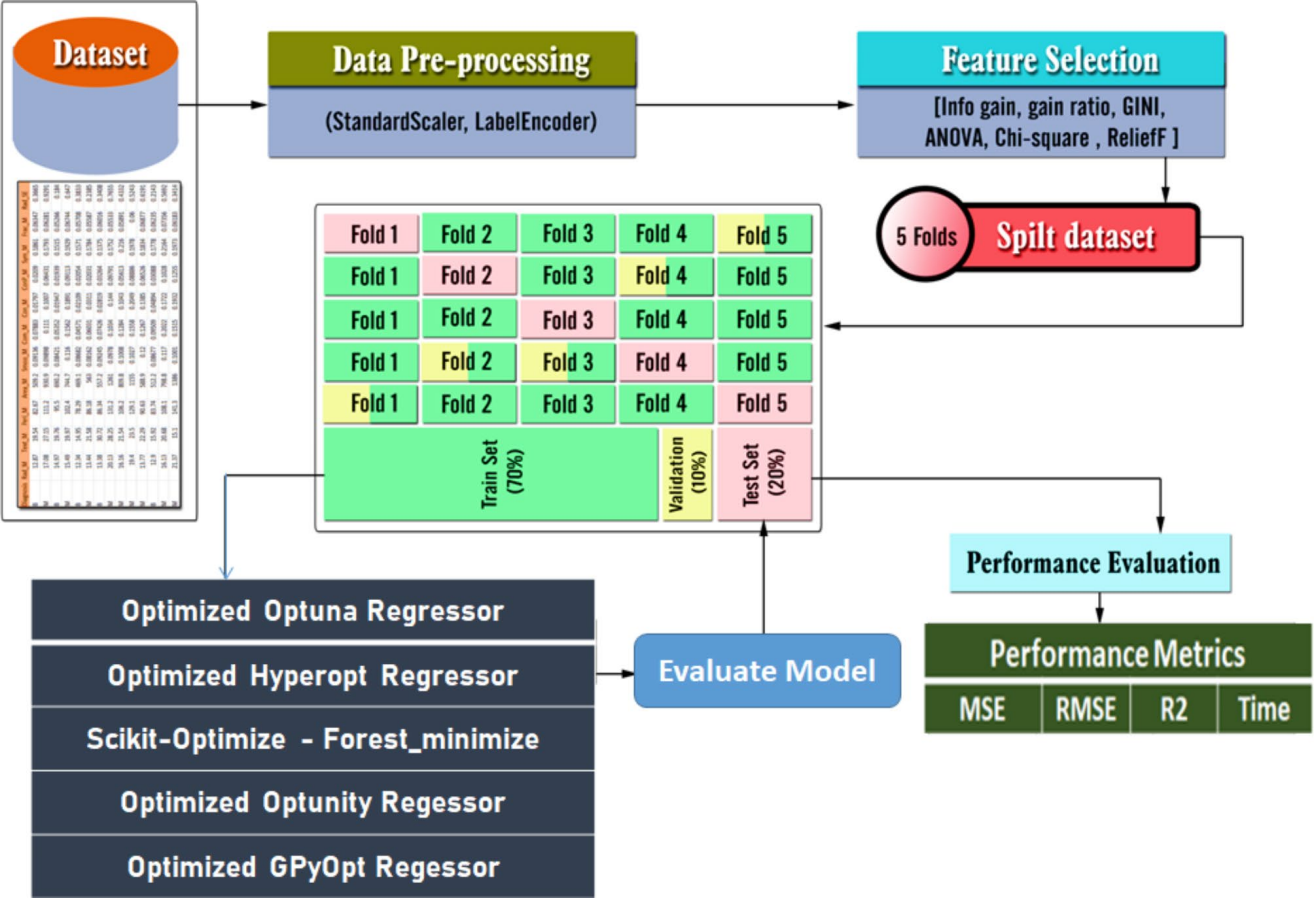
The general framework of the proposed prediction model.

**Fig. 5:**
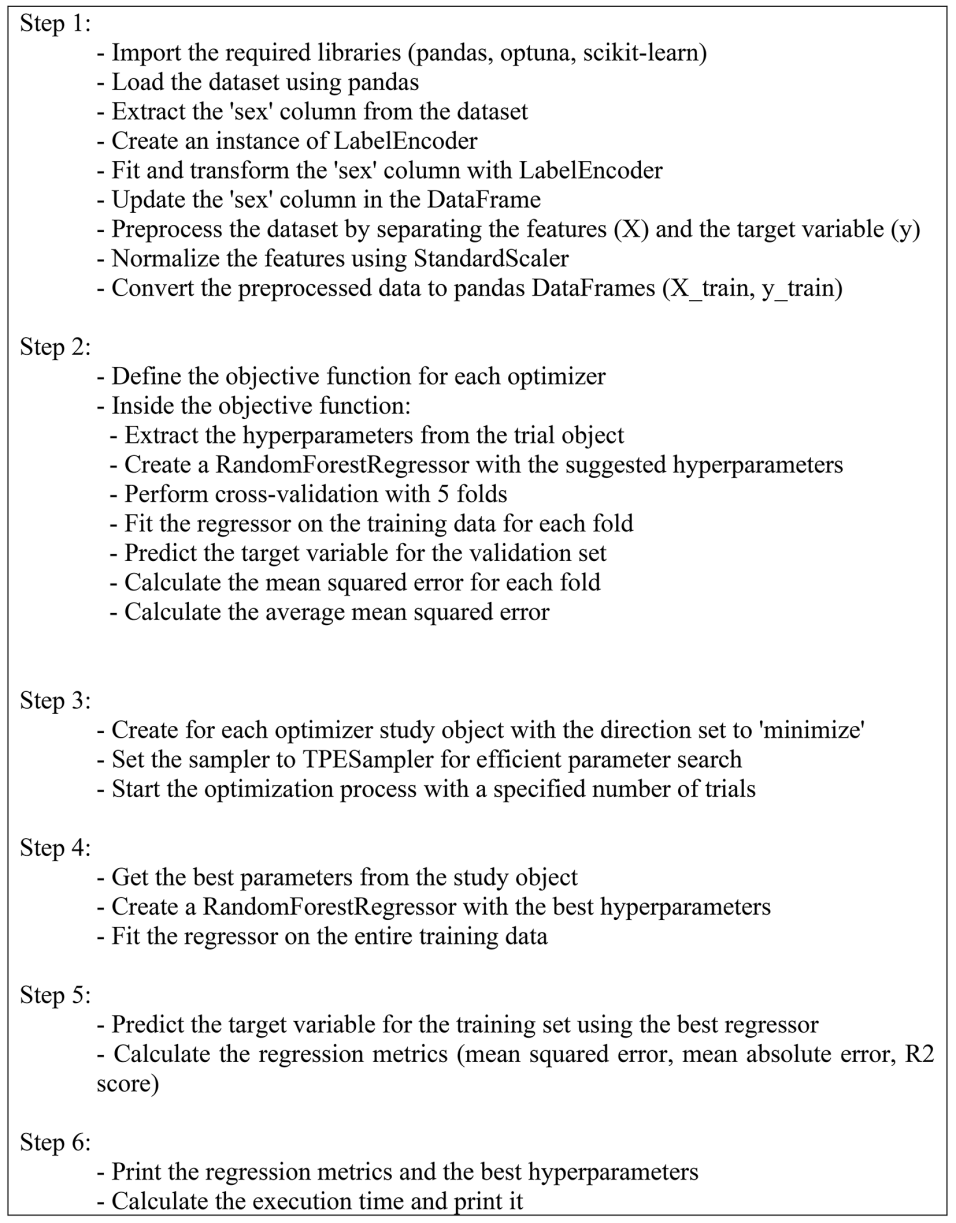
The pseudocode of the proposed optimizers regression model.

Figure 4 provides a visual representation of the proposed prediction model’s overall structure, encompassing the prediction process and performance evaluation metrics. Figure 5 presents the pseudocode for the implemented optimizers.

The key components and processes involved in the model include data loading and preprocessing, data splitting, hyperparameter optimization, model training, model evaluation, and hyperparameter tuning. The objective function for optimization is typically defined as minimizing the mean squared error.

### Evaluation metrics for regression and classification models

#### Evaluation metrics for regression models

The determination coefficient R-square is one of the most common performances used to evaluate the regression model as shown in Eq. (1). On the other hand, the Minimum Acceptable Error (MAE) is shown in Eq. (2), while the Mean Square Error (MSE) is investigated in Eq. (3)^58–60^.

1 $$\:{\text{R}}^{2}=\frac{\sum\:{\left(y-\dot{\widehat{y}}\right)}^{2}}{\sum\:{\left(y-\dot{\overline{y}}\right)}^{2}}$$

2 $$\:\text{M}\text{A}\text{E}=\frac{\sum\:_{i=1}^{n}\left|\widehat{{y}_{i}}-y\right|}{\text{n}}$$

3 $$\:\:\:\text{M}\text{S}\text{E}=\frac{\sum\:_{i=1}^{n}\left|\widehat{{y}_{i}}-{y}_{i}\right|}{\text{n}}$$

Where y is the actual value, $\:\dot{\widehat{\text{y}}}$ is the corresponding predicted value, $\:\dot{\overline{\text{y}}}$ is the mean of the actual values in the set, and ***n*** is the total number of test objects^61^.

## Results and analysis

To evaluate the effectiveness of our machine learning framework, we conducted experiments in this section. The experiments were performed on a computer with a 3 GHz i5 processor, 8GB main memory, and a 64-bit Windows 10 operating system. We used the Python programming language to experiment.

### The results of the proposed regression machine learning technique

**Fig. 6:**
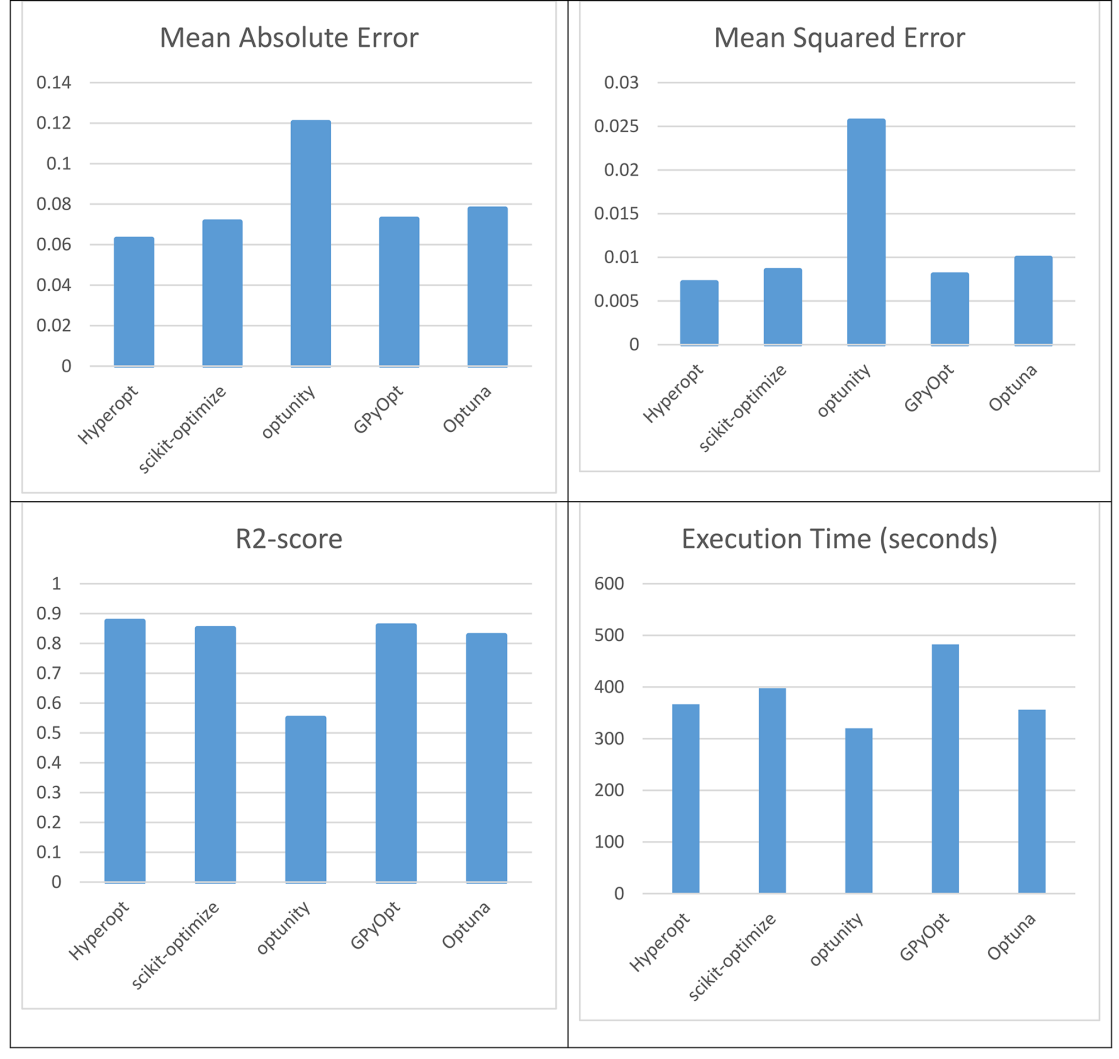
The performance metrics of the optimized regression models of Posttreatment SAS 90.

Tables 5 and 6; Fig. 6 depict the evaluation outcomes of various conventional regression models and optimized regressor models for predicting posttreatment SAS 90, along with their corresponding performance metrics. Table 5 outlines the assessment criteria such as adjusted R-squared, R-squared, root mean squared error (RMSE), and execution time for traditional regression models. In contrast, Table 5 provides insights into the evaluation results of optimized regressor models achieved through different optimization techniques, where the focus lies on mean squared error, mean absolute error, R-squared score, and execution time. Here is an analysis and expansion of the Table:

- Model: This column shows the names of the machine learning models used in the regression task.
- MSE (Mean Squared Error): This column represents the average of the squared differences between the predicted and actual values. A lower value of MSE indicates better performance.
- MAE (Mean Absolute Error): This column represents the average of the absolute differences between the predicted and actual values. A lower value of MAE indicates better performance.
- R2 Score: This column represents the coefficient of determination, which measures the proportion of variance in the target variable that can be explained by the independent variables. A higher value of the R2 Score indicates better performance.
- Time Taken (Seconds): This column represents the amount of time taken by each model to complete the regression task.

**Table 5:** The evaluation of different traditional regression models for Posttreatment SAS 90 to assess their performance.

Model	Adjusted *R*-Squared	*R*-Squared	RMSE	Time Taken (Seconds)
GammaRegressor	0.044256	0.381577	0.110352	0.014992
TweedieRegressor	0.041573	0.379841	0.110507	0.011990
PoissonRegressor	0.040820	0.379354	0.110550	0.013993
LassoLarsCV	0.001930	0.354190	0.112769	0.019426
LarsCV	0.001930	0.354190	0.112769	0.027982
ElasticNetCV	-0.005359	0.349474	0.113180	0.110931
LassoCV	-0.011805	0.345302	0.113542	0.087946
LassoLarsIC	-0.024037	0.337388	0.114227	0.010993
RidgeCV	-0.078959	0.301850	0.117250	0.008995
BayesianRidge	-0.110758	0.281274	0.118965	0.011991
OrthogonalMatchingPursuitCV	-0.111564	0.280753	0.119008	0.021986
BaggingRegressor	-0.168617	0.243836	0.122024	0.026986
Ridge	-0.205619	0.219893	0.123941	0.008994
LinearRegression	-0.227601	0.205670	0.125066	0.010992
Lars	-0.227601	0.205670	0.125066	0.014992
TransformedTargetRegressor	-0.227601	0.205670	0.125066	0.008995
SGDRegressor	-0.294330	0.162492	0.128420	0.010995
RandomForestRegressor	-0.344391	0.130100	0.130880	0.174894
ExtraTreesRegressor	-0.367700	0.115018	0.132009	0.129918
HuberRegressor	-0.447436	0.063424	0.135803	0.022987
OrthogonalMatchingPursuit	-0.453714	0.059362	0.136097	0.010993
PassiveAggressiveRegressor	-0.466074	0.051364	0.136675	0.012993

**Table 6:** The evaluation of optimized regressor models for Posttreatment SAS 90 to assess their performance.

Optimizer	Best Parameters	Mean Squared Error	Mean Absolute Error	R2-score	Execution Time (seconds)
Hyperopt	{‘n_estimators’: 100, ‘max_depth’: 16, ‘min_samples_split’: 2, ‘min_samples_leaf’: 2}	0.0071	0.0627	0.8744	366.5999
scikit-optimize	{‘n_estimators’: 120, ‘max_depth’: 6, ‘min_samples_split’: 4, ‘min_samples_leaf’: 2}	0.0085	0.0712	0.8501	397.7038
optunity	{‘n_estimators’: 472, ‘max_depth’: 16, ‘min_samples_split’: 13, ‘min_samples_leaf’: 9}	0.0256	0.1203	0.5491	320.0005
GPyOpt	{‘n_estimators’: 700, ‘max_depth’: 5, ‘min_samples_split’: 2, ‘min_samples_leaf’: 1}	0.0080	0.0726	0.8589	482.7744
Optuna	{‘n_estimators’: 500, ‘max_depth’: 5, ‘min_samples_split’: 3, ‘min_samples_leaf’: 2}	0.0099	0.0776	0.8265	356.2526

As shown in Tables 5 and 6; Fig. 6:

- Among the traditional regression models, the GammaRegressor, TweedieRegressor, and PoissonRegressor show the highest adjusted R-squared values, indicating a better fit to the data compared to other models. These models explain a small but significant percentage of the variance in the posttreatment SAS 90.
- In contrast, models like PassiveAggressiveRegressor, OrthogonalMatchingPursuit, and HuberRegressor exhibit negative adjusted R-squared values, suggesting that they do not perform well in explaining the variability in the posttreatment SAS 90.
- When considering the mean squared error (MSE) and mean absolute error (MAE), the LassoLarsCV model achieves the lowest values, indicating better accuracy in predicting the posttreatment SAS 90. However, it’s important to note that the MSE and MAE values are relatively high across all models, suggesting that there is still room for improvement in the predictive performance.
- Among the optimization techniques, the scikit-optimize with the forest_minimize method achieves the lowest MSE and MAE values, indicating better accuracy in predicting the posttreatment SAS 90 compared to other optimization techniques. Additionally, it achieves the highest R-squared score, suggesting that it explains a larger portion of the variance in the posttreatment SAS 90.
- However, it’s worth noting that the execution time for the scikit-optimize with the forest_minimize method is the longest, indicating that it takes more time to train and predict with this model compared to other optimization techniques.
- The optimized regressor models using the different optimization algorithms outperformed the traditional regression models in terms of predictive accuracy. Hyperopt showed the best performance among the optimizers, achieving the highest R2-score and the lowest mean squared error and mean absolute error. However, the execution times for the optimized models were relatively longer compared to the traditional models.

### Feature correlations

- The correlation coefficients between distinct features in a dataset are shown in Table 7. The table has been analyzed and expanded as follows:
- First Feature: The name of the first feature being correlated is displayed in this column.
- Second Feature: The name of the second feature being correlated is shown in this column.
- Correlation: The correlation coefficient between the first and second features is represented in this column. A correlation value of one represents a perfect positive correlation, while one shows a perfect negative correlation. A correlation coefficient of 0 shows that there is no relationship.

**Table 7:** Pearson’s correlation of the features.

First Feature	Second Feature	Correlation	First Feature	Second Feature	Correlation
Pretreatment SAS 90	VAS pre	-0.77	BMI	Posttreatment SAS 90	0.144
Posttreatment SAS 90	Pretreatment SAS 90	0.709	VAS pre	age	-0.135
Pretreatment SAS 90	Pretreatment SAS zero	0.616	VAS pre	weight	0.119
Posttreatment SAS 90	Pretreatment SAS zero	0.598	Posttreatment SAS 90	age	0.099
BMI	weight	0.54	BMI	age	-0.091
Posttreatment SAS 90	VAS post	-0.504	BMI	Pretreatment SAS 90	0.09
VAS post	VAS pre	0.478	Pretreatment SAS zero	weight	-0.089
Pretreatment SAS 90	VAS post	-0.445	BMI	Pretreatment SAS zero	0.082
Pretreatment SAS zero	VAS pre	-0.423	Pretreatment SAS 90	weight	-0.049
Posttreatment SAS 90	VAS pre	-0.41	VAS post	weight	-0.044
Pretreatment SAS zero	VAS post	-0.278	BMI	VAS pre	0.04
Pretreatment SAS 90	age	0.234	BMI	VAS post	-0.031
VAS post	age	-0.178	age	weight	0.024
Pretreatment SAS zero	age	0.147	Posttreatment SAS 90	weight	0.017

Based on the Table 7:

- SAS scores (pretreatment, posttreatment, pretreatment zero) are highly correlated with each other, as expected since they measure similar constructs.
- Pain measures (VAS pre, post) are strongly negatively correlated with SAS scores, indicating that increased pain is associated with higher levels of anxiety.
- VAS pre- and post are positively correlated, showing consistency in pain levels before and after treatment.
- BMI has a moderate positive correlation with weight, as one would expect.
- Age has weaker correlations with other variables. It is negatively correlated with pretreatment SAS and VAS pre, but these are small effects.
- Demographic factors like age, weight, and BMI have small correlations with clinical outcomes.

Overall, this analysis confirms:

- Good internal consistency between cognitive/pain measures.
- Expected relationships between BMI/weight.
- Clinical factors are more strongly correlated than demographics.
- Pain inversely linked to cognitive status.
- Weaker influence of basic demographics on main outcomes.

The patterns are consistent with the literature and provide validity for using these variables in further modeling.

### Feature selection

Table 8 provides the selected features for different feature selection methods. Each method aims to identify a subset of features that are considered important for predicting the target variable. The table has been analyzed and expanded as follows^58,62–64^:

- Feature selection technique: The name of the feature selection method used to choose the features is displayed in this column.
- Selected features: The names of the features chosen by the feature selection method are displayed in this column.
- All methods consistently select the main SAS scores (pretreatment, posttreatment) as important features, which align with domain knowledge.
- Clinical features like BMI, age, and weight are selected by many methods. These make intuitive sense as factors influencing outcomes.
- Pain measures (VAS pre, post) also frequently emerge, underscoring their relationship to cognitive scores.
- The SAS scores and clinical features tend to overlap across methods, demonstrating consensus.
- RFE with random forests and feature importance from random forests are model-specific techniques tuned for random forest models.
- Their selections emphasize the SAS scores most, followed by age and BMI - the most predictive features for random forests.

**Table 8:** Feature selection techniques and the most important features.

Feature Selection Method	Selected Features
F-value selector	[‘age’, ‘BMI’, ‘Pretreatment SAS zero’, ‘Pretreatment SAS 90’, ‘VAS pre’]
Mutual information selector	[‘age’, ‘weight’, ‘VAS pre’, ‘VAS post’, ‘Posttreatment SAS 90’]
RFE with logistic regression	[‘Pretreatment SAS zero’, ‘Pretreatment SAS 90’, ‘VAS pre’, ‘VAS post’, ‘Posttreatment SAS 90’]
Select from the model with random forests	[‘Pretreatment SAS zero’, ‘Pretreatment SAS 90’, ‘Posttreatment SAS 90’]
Variance thresholding	[‘age’, ‘weight’, ‘BMI’, ‘VAS pre’, ‘VAS post’]
RFE with random forests	[‘weight’, ‘BMI’, ‘Pretreatment SAS zero’, ‘Pretreatment SAS 90’, ‘Posttreatment SAS 90’]
Feature importance with random forests	[‘Pretreatment SAS zero’, ‘Pretreatment SAS 90’, ‘Posttreatment SAS 90’, ‘BMI’, ‘age’]

Based on the analysis:

- The SAS scores, pain measures, and basic clinical factors can be reliably considered important features.
- Feature selection with random forests (RFE, importance) is best suited since a random forest model will be used.
- Its selections are consistent with domain knowledge and focus on the most predictive attributes for the chosen model type.

Therefore, we would recommend using the features selected by RFE with random forests or feature importance from random forests for the random forest classifier.

## Discussion and future directions

This study leverages machine learning techniques to investigate the complex relationships between patient characteristics and the efficacy of scapular stabilization exercises (SSE) in alleviating non-specific shoulder pain among college students. A crucial distinction must be made regarding the study’s primary objective: rather than comparing the impact of different exercise regimens on shoulder pain, our research focuses on identifying key patient factors that predict the greatest reduction in pain when utilizing SSE as a treatment approach. In essence, our analysis employs machine learning algorithms to uncover the most influential predictors (e.g., sex, weight, BMI, pre-treatment Self-Appraisal Scale [SAS] scores) that determine the likelihood of successful pain reduction with SSE. This approach enables the development of personalized treatment strategies, tailored to the specific profiles of patients who are most likely to benefit from scapular stabilization exercises.

### Key findings and implications

1. Predictive Modeling for Personalized Treatment: Our machine learning models successfully identified significant correlations between patient characteristics and the efficacy of SSE in reducing shoulder pain. These findings facilitate the creation of personalized treatment plans, enhancing the potential for positive outcomes.
2. Patient Profiling for Optimal SSE Response: The study’s results provide valuable insights into the types of patients who are most suited for scapular stabilization exercises as a treatment for non-specific shoulder pain. For instance, [insert specific patient characteristics identified by the study, e.g., “patients with a lower BMI and higher pre-treatment SAS scores”] demonstrated a more significant reduction in pain when undergoing SSE.
3. Clinical Utility and Future Directions: By integrating these predictive models into clinical practice, healthcare professionals can make more informed decisions when recommending scapular stabilization exercises for patients with non-specific shoulder pain. Future research should focus on validating these findings across broader populations and exploring the application of similar machine learning approaches to other musculoskeletal conditions.

### Clarification on study objectives and interpretation

To reiterate, this manuscript does not aim to compare the effectiveness of different exercise regimens but rather to elucidate the patient factors that predict successful outcomes with scapular stabilization exercises. The discussion and interpretation of our results are grounded in this core objective, providing a nuanced understanding of how SSE can be optimized for specific patient populations suffering from non-specific shoulder pain.

Furthermore, the substantial execution times associated with some optimization techniques, such as optunity and GPyOpt, emphasize the practical considerations and trade-offs involved in implementing these methodologies in real-world clinical settings. Balancing computational efficiency with predictive accuracy remains a critical consideration for the seamless integration of machine learning-based interventions into routine musculoskeletal rehabilitation protocols.

In light of our findings on predicting successful outcomes with scapular stabilization exercises (SSE) for non-specific shoulder pain, two key avenues for future research emerge:

- Validation and Generalizability: Validate the predictive models developed in this study across diverse populations to enhance their generalizability and applicability in various clinical settings.
- Integration with Clinical Practice: Investigate the feasibility and effectiveness of integrating these predictive models into clinical decision-support systems to personalize SSE treatment plans for patients with non-specific shoulder pain.

### Limitations

Despite the significant contributions and promising results of our study on predicting abdominal fat dynamics in the context of cavitation treatments, several limitations should be acknowledged:

1. Limited Sample Size: The study utilized a comprehensive dataset; however, the sample size may still be relatively small. A larger sample size would enhance the statistical power and generalizability of the findings. The limited sample size may also restrict the exploration of potential subgroups or variations within the population.
2. Potential Bias and Confounding Factors: The dataset used in the study may contain inherent biases or confounding factors that could influence the results. Unaccounted variables, such as age, gender, specific medical conditions, and concurrent treatments, may impact the fat dynamics and introduce potential bias into the predictive models.
3. In this study were collected retrospectively, which may introduce limitations and potential biases inherent in retrospective analyses. Prospective studies with standardized protocols and data collection methods would provide more robust and reliable evidence.
4. Lack of Patient-reported Outcomes: The study primarily relied on objective measurements of fat dynamics and did not incorporate patient-reported outcomes, such as satisfaction, quality of life, or subjective perception of body contouring. Including patient-reported outcomes would provide a more holistic assessment of the treatment effects.

## Conclusions

This study utilized an extensive array of machine learning models and optimization techniques to predict the impact of scapular stabilization exercises on non-specific shoulder pain among college students. The results underscore the critical role of scapular stabilization exercise in improving shoulder function and reducing pain, highlighting the potential of machine learning in optimizing therapeutic strategies for musculoskeletal health management. Among the diverse regression models employed, the Gamma Regressor, Tweedie Regressor, and Poisson Regressor demonstrated the highest adjusted R-squared values, indicating their relatively stronger predictive performance in capturing the relationships between exercise protocols and pain reduction. Furthermore, the scikit-optimize optimization approach exhibited the most promising results, yielding the lowest mean squared error and mean absolute error, alongside the highest R-squared score, signifying its effectiveness in fine-tuning the exercise parameters for optimal outcomes. The findings suggest that a data-driven approach, facilitated by machine learning techniques, can significantly enhance the precision and efficacy of scapular stabilization exercise regimens, ultimately leading to improved shoulder health and functionality among college students. By leveraging advanced optimization methodologies, such as scikit-optimize, the study emphasizes the importance of customizing exercise protocols based on individual biomechanical profiles, thus addressing the diverse needs and concerns associated with non-specific shoulder pain in this specific demographic.

## Data Availability

Data and code availability statement. The dataset and code used in this study are public and all test data are available at this portal (https://github.com/tarekhemdan/SAS).
